# ICTV Virus Taxonomy Profile: Anelloviridae 2026

**DOI:** 10.1099/jgv.0.002222

**Published:** 2026-03-30

**Authors:** Simona Kraberger, Tanja Opriessnig, Fabrizio Maggi, Vladimir Celer, Hiroaki Okamoto, Lia van der Hoek, Philippe Biagini, Mart Krupovic, Arvind Varsani

**Affiliations:** 1 The Biodesign Center for Fundamental and Applied Microbiomics, Center for Evolution and Medicine, School of Life Sciences, Arizona State University, 1001 S. McAllister Ave, Tempe, AZ 85287-5001, USA; 2 Moredun Research Institute, Pentland Science Park, Bush Loan, EH26 0PZ Penicuik, UK; 3 Virology and Laboratories of Biosecurity, National Institute for Infectious Diseases Lazzaro Spallanzani – IRCCS, 00149 Rome, Italy; 4 Faculty of Veterinary Medicine, University of Veterinary Sciences Brno, Palackeho 1946, 612 42 Brno, Czechia; 5 Division of Virology, Department of Infection and Immunity, Jichi Medical University School of Medicine, 3311-1 Yakushiji, Shimotsuke-shi, Tochigi 329-0498, Japan; 6 Laboratory of Experimental Virology, Department of Medical Microbiology and Infection Prevention, Amsterdam UMC, Meibergdreef 9, 1105 AZ Amsterdam, the Netherlands; 7 Equipe GENGLOBE, UMR 7268 ADES, Aix-Marseille Université, CNRS, EFS, 27 Bd. Jean Moulin, 13005 Marseille, France; 8 Institut Pasteur, Université Paris Cité, Cell Biology and Virology of Archaea Unit, 25 rue du Dr Roux, 75015 Paris, France

**Keywords:** *Anelloviridae*, ICTV Report, taxonomy

## Abstract

The family *Anelloviridae* includes viruses with circular, negative-sense, ssDNA genomes of 1.7–3.9 kb. Anelloviruses form non-enveloped icosahedral virions constructed of the ORF1 protein, which adopts the single jelly-roll structural fold. Anelloviruses have been isolated from a broad range of birds and mammals, including humans, but are not associated with pathogenicity, except for members of the genus *Gyrovirus*. This is a summary of the International Committee on Taxonomy of Viruses (ICTV) Report on the family *Anelloviridae*, which is available at www.ictv.global/report/anelloviridae.

## Virion

**Fig. 1.**
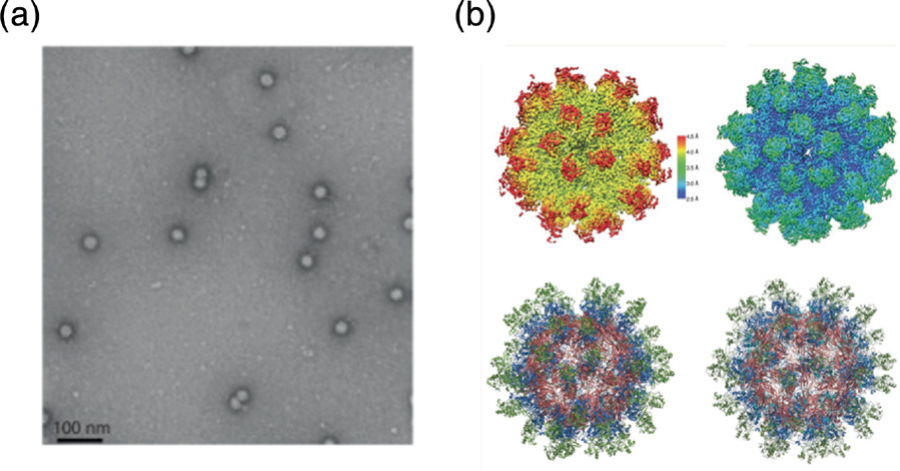
Anellovirus particles. (a) Negative-stained electron microscopy image of anellovirus-like particles generated from TTMV-LY1 constructs. (b) Cryo-EM structures of anellovirus-like particles (Images from Ring Therapeutics, Inc., [2]).

Anellovirids form non-enveloped, icosahedral (*T=*1) capsids, 30–32 nm in diameter (Table 1). The capsid is built from 60 copies of the capsid protein (encoded by *orf1*), which adopts the single jelly-roll structural fold [1]. In some anellovirids, ORF1 protein contains a large insertion within the jelly-roll domain corresponding to a (spike) domain, which projects away from the capsid surface, forming crown-like structures around the fivefold symmetry axes (Fig. 1) [2].

**Table 1.** Characteristics of viruses in the family *Anelloviridae*

Example	torque teno virus 1 (AB041007), species *Alphatorquevirus homin1*
Virion	Non-enveloped, icosahedral (*T=*1) capsid, 30–32 nm in diameter
Genome	Circular molecule of negative-sense ssDNA (1.7–3.9 kb)
Replication	Nuclear
Translation	From multiple, spliced mRNAs
Host range	Mammals, birds
Taxonomy	Realm *Floreoviria*, kingdom *Shotokuvirae*, phylum *Commensaviricota*, class *Cardeaviricetes*, order *Sanitavirales*: >35 genera, >240 species

## Genome

**Fig. 2.**
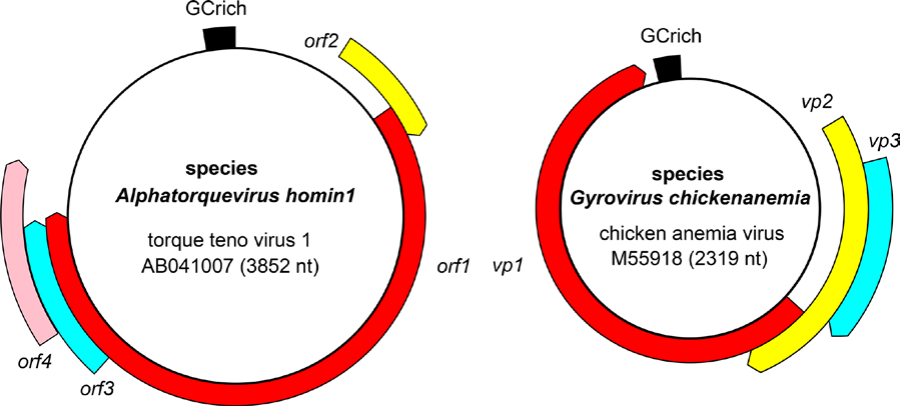
Organization of representative anellovirid genomes.

The genomes of anelloviruses are non-segmented, negative-sense circular ssDNA molecules of 1.7–3.9 kb [3,4]. The putative non-coding region generally has a high G+C content and is postulated to form secondary structures composed of stem loops. Most anelloviruses have three genes, known as *orf1* (*vp1* in gyroviruses), *orf2* (*vp2* in gyroviruses) and *orf3* (*vp3* in gyroviruses), although certain anelloviruses may carry additional ORFs (Fig. 2). The largest gene is *orf1*, which codes for the capsid protein. The proportion of the genome occupied by *orf1* varies widely between species [4]. The N-terminal region of ORF1 protein is largely unstructured and rich in arginine residues; it is postulated to be important for nuclear localization, signalling and binding to the viral genome [5]. This region is followed by a jelly-roll domain, which plays a key role in capsid formation, and the spike domain, predicted to be important for host recognition and/or immune evasion [1,2]. The other ORFs typically overlap *orf1* at both 5′- and 3′-termini.

## Replication

Knowledge of genome expression and replication mechanisms remains limited, mainly owing to the lack of an efficient cell culture propagation system. At least three mRNAs of different sizes are transcribed from the putative circular double-stranded replicative form of the anellovirus genome. Recent evidence suggests that the ORF2/3 product plays a role in initiating viral genome replication through a recombination-dependent mechanism with the aid of host-encoded replication factors [6].

## Pathogenicity

Infection is highly prevalent and often chronic. No direct links to disease have been established for anelloviruses other than chicken anaemia virus (genus *Gyrovirus*), which is associated with immunosuppressive disease in chickens, resulting in a range of clinical outcomes including weight loss, anaemia and intramuscular haemorrhaging [7]. By contrast, in mammalian hosts, the nearly ubiquitous presence of anelloviruses without apparent pathogenesis has led to the hypothesis that these viruses are commensals [8].

## Taxonomy

Current taxonomy: ictv.global/taxonomy. The family *Anelloviridae* includes >35 genera and >240 species. Members of all but one genus are associated with mammals (or blood-feeding insects); members of the remaining genus, *Gyrovirus*, include members identified in birds or faecal samples from other animals.

## Resources

Full ICTV Report on the family *Anelloviridae*: www.ictv.global/report/anelloviridae.
